# Alterations in large-scale resting-state network nodes following transcranial focused ultrasound of deep brain structures

**DOI:** 10.3389/fnhum.2024.1486770

**Published:** 2024-12-04

**Authors:** Stephanie M. Gorka, Jagan Jimmy, Katherine Koning, K. Luan Phan, Natalie Rotstein, Bianca Hoang-Dang, Sabrina Halavi, Norman Spivak, Martin M. Monti, Nicco Reggente, Susan Y. Bookheimer, Taylor P. Kuhn

**Affiliations:** ^1^Department of Psychiatry and Behavioral Health, Wexner Medical Center, The Ohio State University, Columbus, OH, United States; ^2^Department of Psychiatry and Biobehavioral Sciences, The University of California, Los Angeles, Los Angeles, CA, United States; ^3^Department of Psychology, The University of California, Los Angeles, Los Angeles, CA, United States; ^4^Institute for Advanced Consciousness Studies, Santa Monica, CA, United States

**Keywords:** transcranial focused ultrasound, resting-state functional connectivity, amygdala, entorhinal cortex, salience network (SN), default mode network

## Abstract

**Background:**

Low-intensity transcranial focused ultrasound (tFUS) is a brain stimulation approach that holds promise for the treatment of brain-based disorders. Studies in humans have shown that tFUS can successfully modulate perfusion in focal sonication targets, including the amygdala; however, limited research has explored how tFUS impacts large-scale neural networks.

**Objective:**

The aim of the current study was to address this gap and examine changes in resting-state connectivity between large-scale network nodes using a randomized, double-blind, within-subjects crossover study design.

**Methods:**

Healthy adults (*n =* 18) completed two tFUS sessions, 14 days apart. Each session included tFUS of either the right amygdala or the left entorhinal cortex (ErC). The inclusion of two active targets allowed for within-subjects comparisons as a function of the locus of sonication. Resting-state functional magnetic resonance imaging was collected before and after each tFUS session.

**Results:**

tFUS altered resting-state functional connectivity (rsFC) within and between rs-network nodes. Pre-to-post sonication of the right amygdala modulated connectivity within nodes of the salience network (SAN) and between nodes of the SAN and the default mode network (DMN) and frontoparietal network (FRP). A decrease in SAN to FPN connectivity was specific to the amygdala target. Pre-to-post sonication of the left ErC modulated connectivity between the dorsal attention network (DAN) and FPN and DMN. An increase in DAN to DMN connectivity was specific to the ErC target.

**Conclusion:**

These preliminary findings may suggest that tFUS induces neuroplastic changes beyond the immediate sonication target. Additional studies are needed to determine the long-term stability of these effects.

## Introduction

Transcranial focused ultrasound (tFUS) is a novel approach to brain stimulation that holds immense promise for the treatment of brain-based disorders (Pasquinelli et al., 2019; Stern et al., 2021). At high intensities, tFUS can be used to locally ablate brain tissue and provide irreversible treatment (di Biase et al., 2021; Moosa et al., 2019), and at low intensities, tFUS can be used to transiently modulate the functioning of specific brain regions by non-invasively applying acoustic energy (Tyler et al., 2008; Dell'Italia et al., 2022). Unlike other neuromodulation approaches, low-intensity tFUS can reach deep brain structures with high spatial precision to inhibit or enhance neural activation (Romanella et al., 2020). By manipulating ultrasound parameters, it is also possible to change brain function without causing tissue damage (Spivak et al., 2022). Low-intensity tFUS therefore overcomes many of the limitations of existing neuromodulation techniques and is being increasingly explored as a novel treatment approach for a variety of neurological and psychiatric disorders.

tFUS has been applied to a variety of brain regions, including somatosensory and visual cortices, insula, thalamus, and striatum (Legon, 2021; Monti et al., 2016; Cain et al., 2021). Recent studies have also begun to target the amygdala, a brain region known to mediate threat and emotion processing (Ohman, 2005; Folloni et al., 2019; Kuhn et al., 2023; Chou et al., 2024). Three decades of neuroimaging research indicate that hyperactivity of the amygdala is involved in the pathophysiology of internalizing disorders, including anxiety and post-traumatic stress disorder (Ressler and Nemeroff, 2000; Gerin et al., 2019). Early studies show that tFUS of the amygdala can modulate amygdala perfusion and alleviate symptoms of anxiety (Zielinski et al., 2021; Mahdavi et al., 2023). For example, a case study of an individual with treatment-resistant generalized anxiety disorder (trGAD) demonstrated that tFUS of the amygdala resulted in immediate decreases in anxiety symptoms (Zielinski et al., 2021). In a larger cohort of patients with trGAD, eight weekly tFUS sessions targeting the amygdala similarly resulted in significant decreases in anxiety symptoms, with 64% of patients achieving clinically significant benefit (Mahdavi et al., 2023). Converging research therefore suggests that tFUS has therapeutic potential and the safety, efficacy, and neural mechanisms of tFUS should be further explored.

How tFUS modulates neural processes is still unclear (Dell'Italia et al., 2022). Studies indicate that tFUS increases or decreases blood oxygen level-dependent (BOLD) signal depending on sonication parameters (Kuhn et al., 2023; Chou et al., 2024). Indeed, a study by our group found that tFUS of the amygdala selectively increased perfusion in the right amygdala but not the left amygdala without engaging the auditory cortex (Kuhn et al., 2023). How these focal changes influence the rest of the brain is still an area under active investigation. Using BOLD data collected simultaneously with the tFUS sonication experiment, we found decreases in functional connectivity between the right amygdala and posterior cingulate, anterior cingulate, medial prefrontal, and posterior parietal regions in a sample of healthy older adults (Kuhn et al., 2023). A separate study by Chou et al. (2024) examined changes in resting-state functional connectivity (rsFC) in healthy adults before and after active or sham tFUS of the left amygdala. Chou et al. (2024) reported that active tFUS resulted in decreased amygdala-insula and amygdala-hippocampal rsFC and increased amygdala-ventromedial prefrontal cortex rsFC. Meanwhile, a recent study by Peng et al. (2024) found that tFUS of the left nucleus accumbens was associated with increased rsFC between the nucleus accumbens and the medial prefrontal cortex.

Existing studies provide important initial evidence that tFUS changes FC between the sonication target and other areas of the brain. However, no study to date has directly examined tFUS-related changes to neural networks beyond the target. The brain is organized into multiple distributed (large-scale) systems, including the default mode network (DMN), dorsal attention network (DAN), frontoparietal network (FPN), and salience network (SN) (Yeo et al., 2011). Synchronized activity within each network is observed under resting conditions and posited to underlie specific cognitive-affective functions, including interception and attentional control (Hellyer et al., 2014). Converging evidence from other techniques suggests that neuromodulation can affect brain networks beyond the focal stimulation target (To et al., 2018; Pini et al., 2018; Santarnecchi et al., 2018). Thus, in order to fully elucidate the neuroplastic changes associated with tFUS, it is necessary to investigate “down-steam” changes within and between well-characterized intrinsic neural networks.

In the present study, we collected resting-state BOLD fMRI before and after tFUS to characterize changes in rsFC. We used a sonication paradigm adapted from previous studies to inhibit/disrupt amygdala activity (Spivak et al., 2022; Folloni et al., 2019). We also included a second (within-subjects) target as an active regional comparison—the entorhinal cortex (ErC). The ErC is implicated in memory formation (Montchal et al., 2019) and hypoactive in diseases characterized by memory disturbance, e.g., Alzheimer’s disease and mild cognitive impairment (Igarashi, 2023). In a randomized, double-blind, within-subjects crossover study design, we enrolled participants to complete two tFUS sessions, separated by a 14-day between-session window. The sonication paradigm for left ErC was hypothesized to excite/stimulate and therefore increase ErC activity and connectivity (Spivak et al., 2022; Dell'Italia et al., 2022). In a previously published study of these participants, we demonstrated that the sonication protocols selectively increased perfusion in the targeted region, but not in the contralateral homolog or either of the bilateral control regions (Kuhn et al., 2023). Data were collected and examined blindly to determine the target-specific network changes in rsFC pre-to-post sonication. We broadly expected decreased rsFC connectivity within and between network nodes following the sonication of the amygdala target and increased rsFC connectivity within and between network nodes following the sonication of the ErC target based on the specific tFUS parameters.

## Methods

### Participants

A total of 20 healthy adults were enrolled in the study: 17 individuals completed both experimental sessions (4 scans), 18 individuals completed the amygdala tFUS session (2 scans), and 19 individuals completed the ErC tFUS session (2 scans). All participants were required to be between 30 and 85 years of age, right-handed, and proficient in English. Exclusionary criteria included contraindications for MRI, history of serious head injury, history of any major psychiatric illness requiring treatment, and history of any major neurological disorder (e.g., epilepsy) or serious illness (e.g., cancer). The age range was selected to obtain a sample representative of healthy adult aging to ultimately expand this line of work into this understudied population. In individuals 60 years and older, participants were required to score > 30 on the Telephone Interview for Cognitive Status-Modified (TICS-M) (REF) to ensure the absence of cognitive impairment.

The sample had an average age of 61.38 years (7.75; range = 48–79), with 56% female participants. The ethnic distribution was 37% Caucasian American, 31% Latinx American, 19% African American, and 13% Asian American. All procedures were in accordance with the Declaration of Helsinki and approved by the University of California, Los Angeles (UCLA) Institutional Review Board prior to enrollment. All participants provided written informed consent.

### Procedures

The study was a double-blind randomized, within-subjects crossover clinical trial (NCT03717922). Each participant completed two experimental sessions that were separated by exactly 2 weeks. Resting-state FC was collected pre-tFUS and post-tFUS. Target order was randomized: one session targeted the right amygdala (experimental target) and the other session targeted the left entorhinal cortex (ErC; control target). Participants and study staff performing statistical analyses were blinded to the target (1 vs. 2). Following each lab session, participants were followed for three continuous days to assess possible adverse events. No adverse events occurred during the study, and no negative reactions were reported, including physical discomfort or heightened anxiety.

### MRI-guided tFUS sonication protocol

The details of our sonication protocol are published elsewhere (Kuhn et al., 2023). In brief, sonications were delivered using a single-element transducer placed above the ear at the temporal window and targeted using real-time structural MRI navigation inside the scanner. The amygdala sonication protocol was designed to decrease or inhibit neural activity, drawing in part from the Foloni study in macaques (Folloni et al., 2019). This protocol was selected based on the hypothesis that inhibition of amygdala activity may guide additional research on tFUS clinical applications in anxiety disorders. The ErC sonication protocol was designed to increase or excite neural activity and was based in part on collaborators’ work in ErC DBS (Suthana et al., 2012), which suggested that stimulation of the ErC may improve learning and memory. Both paradigms used a 5% duty cycle with 10 cycles of 30 s on, 30 s off, for a total of 5 min of non-consecutive tFUS. The amygdala target included a 5 ms pulse width repeated at a 10 Hz pulse repetition frequency (PRF), while the ErC target included a 0.5-ms pulse width repeated at a 100 Hz PRF. Across both sessions, the fundamental frequency was 0.65 MHz and the I_spta.3_ was 720 mW/cm^2^.

tFUS was performed inside the scanner. A 30-s SCOUT imaging sequence was used to visualize the transducer and its orthogonal line into the brain from the interface of the transducer and gel pad. The focal sonication depth was 65 mm or 55 mm (BrainSonix Corp., Sherman Oaks, CA, USA; Schafer et al., 2020) depending on each participant’s anatomical requirements to reach the desired brain target. The transducer was manually moved, as necessary, to correct its position for the appropriate target. The focus of the targeting line was either the centromedian aspect of the amygdala or the interface of the ErC and the perforant pathway. As reported in Kuhn et al. (2023), partial volume corrected arterial spin labeling MRI demonstrated increased perfusion in the region of the brain targeted by tFUS (amygdala or ErC) as compared to the control region.

### Resting state and structural data acquisition

The MRI data were collected using a 3 T Siemens MAGNETOM Prisma fit scanner (Siemens Medical Solution, Erlangen, Germany) located at the UCLA Center for Cognitive Neuroscience. A 20-channel head coil was used for all acquisition sequences to accommodate the tFUS transducer. Resting-state BOLD data were collected before and after tFUS using a GRE EPI sequence with the following acquisition parameters: TR = 800 ms, TE = 37 ms, flip angle = 52°, FOV = 208 mm (AP and RL) × 144 mm (FH), voxel size = 2.0 × 2.0 × 2.0 mm, slice thickness = 2.0 mm, slice count = 72 slices, phase encoding directio*n =* AP, multi-band acceleration factor = 8, acquisition mode = interleaved, and total volumes = 488; thus, each resting-state run had a total run time of 390.40 s. Framewise Integrated Real-time MRI Monitoring (FIRMM) (Dosenbach et al., 2017) was used during the collection of all BOLD data to monitor participant motion.

To correct for geometric distortions, opposite phase-encoded spin-echo field map images were collected with the following parameters: phase encoding directions = AP and PA, TR = 8,000 ms, TE = 66 ms, voxel size = 2.0 × 2.0 × 2.0 mm, FOV = 208 mm (AP and RL) and 144 mm (FH), slice thickness = 2.0 mm, flip angle = 90°, and refocus flip angle = 180°, and single-band acquisition. Additionally, structural MP-RAGE T1-weighted scans were acquired with the following parameters: orientatio*n =* sagittal, slices = 176, voxel size = 1.0 × 1.0 × 1.0 mm, slice thickness = 1.0 mm, TR = 2,300 ms, TE = 2.98 ms, TI = 900 ms, flip angle = 9°, FOV = 256 mm (FH) and 248 mm (AP), and acceleration factor = 2 (GRAPPA).

### Data preprocessing

All MRI data processing and analyses were carried out using the storage and computing service provided by the Ohio Supercomputer Center. The BOLD data were head motion-corrected using McFlirt (FSL v6.0.4). Distortion correction was estimated from opposite phase-encoded spin-echo images using FSL’s TOPUP. After which, the BOLD data were co-registered to the subject’s T1w using boundary-based registration as implemented by bbregister (FreeSurfer v7.1.1) with 12 degrees of freedom. The BOLD data were then brought to the MNI template space by applying the T1w-to-MNI template (MNI152NLin2009cAsym) warp computed by antsRegistration (ANTs v2.3.5). To minimize smoothing effects, all the transformations were concatenated and applied in a single step to the BOLD volumes using antsApplyTransforms (ANTS v2.3.5); the images were sampled to the final space using Lanczos interpolation.

The preprocessed BOLD data were further denoised using the CONN toolbox (v21a). Effects of nuisance variables such as signals from white matter and cerebrospinal areas, movement parameters and their first derivative, and outlier scans were regressed out using the default implementation in CONN. A band-pass filter of 0.008–0.09 Hz and linear detrending were also applied. Mean framewise displacement (FD) was calculated for each session, pre- (amygdala: 0.34 ± 0.17; ErC: 0.33 ± 0.13) and post-sonication (amygdala: 0.32 ± 0.12; ErC: 0.38 ± 0.20). Within-subjects, there were no differences in mean FD between pre- and post-sonication resting-state scans during the amygdala target (*t*[17] = 1.25, *p* = 0.227) and the ErC target (*t*[17] = −1.73, *p* = 0.100) sessions.

Nineteen regions of interest (ROI) were chosen from CONN’s network cortical ROI atlas to be included in our analyses to estimate ROI-to-ROI functional connectivity. Specifically, the ROIs were derived from an independent-component analysis of resting-state data from the Human Connectome Project (Whitfield-Gabrieli and Nieto-Castanon, 2012). We used these ROIs because they are standardized and easily accessible, which increases the reproducibility of the analyses. Although a wider set of network ROIs are available in the CONN Toolbox, we focused on the regions and nodes most often implicated in amygdala and/or ErC research. These regions included four ROIs from the default mode network (DM), seven ROIs from the salience network (SA), four ROIs from the frontal–parietal network (FP), and four ROIs from the dorsal attention network (DA). The individual ROIs are listed in Table 1. A total of 171 ROI-to-ROI functional connections were estimated between the chosen regions. Here, ROI-to-ROI functional connectivity was estimated as the Fisher transformed bivariate correlation coefficient between any given pair of ROI BOLD time series.

**Table 1:** Regions of interest used in the primary analyses.

Network	Region	Voxel size (mm^3^)
Dorsal attention	IntraParietal sulcus (IPS) (R) (39, −42, 54)	25,096
Dorsal attention	IntraParietal sulcus (IPS) (L) (−39, −43, 52)	26,280
Dorsal attention	Frontal eye field (FEF) (R) (30, −6, 64)	432
Dorsal attention	Frontal eye field (FEF) (L) (−27, −9, 64)	704
Salience	SupraMarginal gyrus (SMG) (L) (−60, −39, 31)	1864
Salience	SupraMarginal gyrus (SMG) (R) (62, −35, 32)	2,272
Salience	Anterior insula (AIC) (R) (47, 14, 0)	3,104
Salience	Anterior insula (AIC) (L) (−44, 13, 1)	3,568
Salience	Anterior cingulate (ACC) (0, 22, 35)	8,504
Salience	Rostral prefrontal cortex (RPFC) (L) (−32, 45, 27)	9,328
Salience	Rostral prefrontal cortex (RPFC) (R) (32, 46, 27)	4,648
Default mode	Medial prefrontal cortex (MPFC) (1, 55, −3)	10,768
Default mode	Lateral parietal (LP) (L) (−39, −77, 33)	8,328
Default mode	Lateral parietal (LP) (R) (47, −67, 29)	10,608
Default mode	Precuneus cortex (PCC) (1, −61, 38)	38,664
Fronto parietal	Posterior parietal cortex (PPC) (L) (−46, −58, 49)	6,656
Fronto parietal	Posterior parietal cortex (PPC) (R) (52, −52, 45)	6,696
Fronto parietal	Lateral prefrontal cortex (LPFC) (L) (−43, 33, 28)	13,624
Fronto parietal	Lateral prefrontal cortex (LPFC) (R) (41, 38, 30)	14,064

To assess the overall reliability of the resting-state scans, the intra-class correlation coefficient (ICC) was computed between the two pre-LIFU scans using pairwise connectivity values between all selected ROIs. The pingouin Python package was used to compute ICC, specifically ICC (3, 1), also known as the single fixed rater method. Participant IDs were used as the target variables, and scanning session IDs were used as the rater variables for ICC computation. The resting-state scans showed good reliability between the two pre-LIFU scans: ICC (3, 1) = 0.733, *F*_(16, 16)_ = 6.51, *p* = 0.000264, and 95% CI [0.40, 0.89].

### Data analysis plan

We first performed a within-subjects paired samples *t*-test on all ROI-to-ROI connections for the experimental amygdala target and control ErC target, separately. Given that this was a preliminary investigation, to balance between statistical power and Type I and II error, we set a threshold of *p* < 0.01, uncorrected for multiple comparisons. Pre-to-post changes in rsFC that were found to be statistically significant (for either target) were then examined for target specificity. Extracted parameter estimates were entered into a target (amygdala vs. ErC) by time (pre vs. post) within-subjects repeated measures ANOVA. Significant two-way interactions were then followed up by performing standard within-subjects comparisons.

## Results

### Amygdala target: network changes pre-to-post sonication

**Figure 1:**
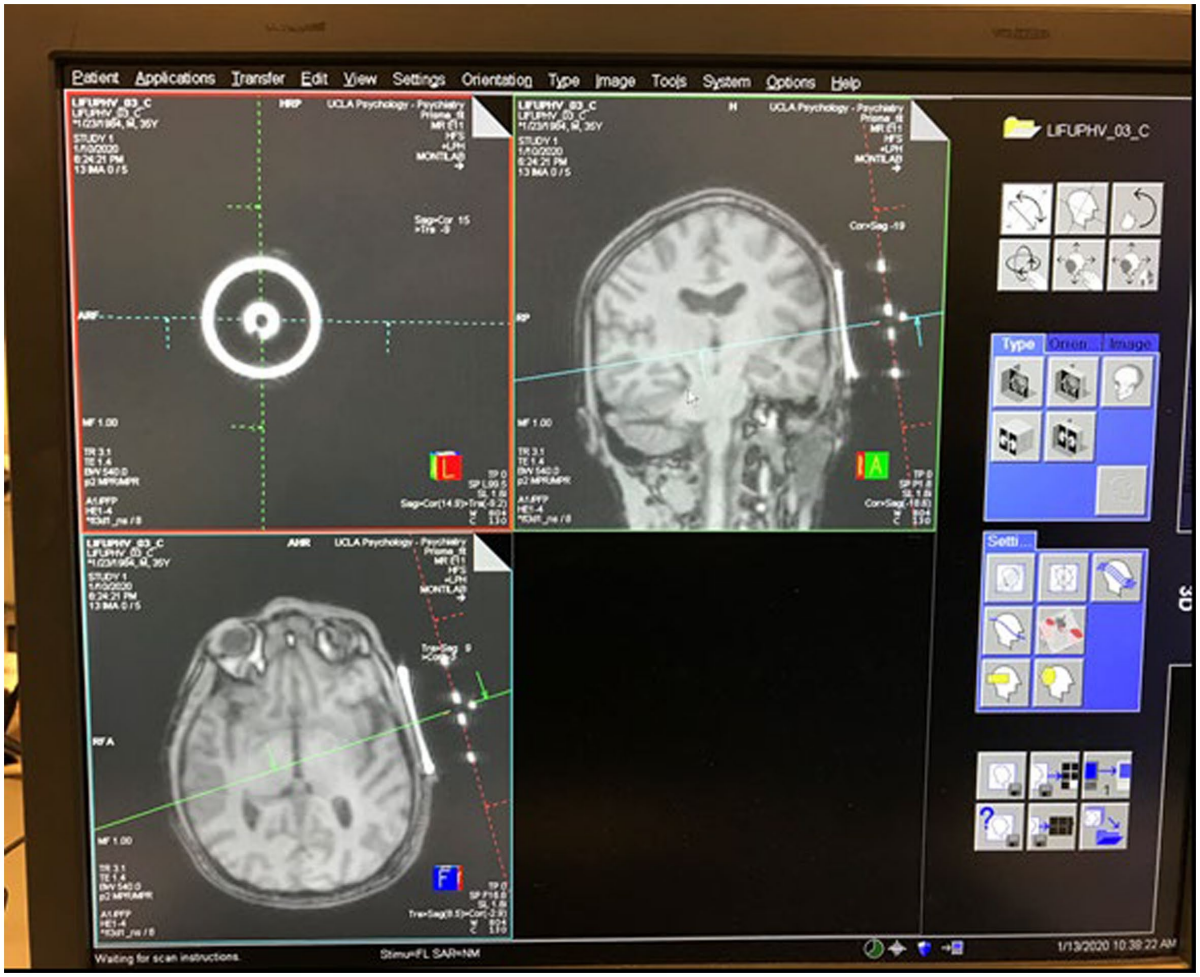
Illustration of the MRI-guided set-up.

The results revealed significant change in ROI-to-ROI rsFC between several regions, across different networks. The results are displayed in Figure 1. The following pre-to-post sonication changes in connectivity were observed: (1) increase in the left rostral prefrontal cortex (RPFC; SA network) to the precuneus cortex (DMN network; *t*[17] = 3.99, *p* = 0.0009); (2) increase in the right anterior insula cortex (AIC; SA network) to the precuneus cortex (DM network; *t*[17] = 3.52, *p* = 0.0026); (3) increase in the right AIC (SA network) to the left RPFC (SA network; *t*[17] = −3.10, *p* = 0.0065); and (4) decrease in the right RPFC (SA network) to the right lateral prefrontal cortex (LPFC; FP network; *t*[17] = −3.03, *p* = 0.0075).

### ErC target: network changes pre-to-post sonication

**Figure 2:**
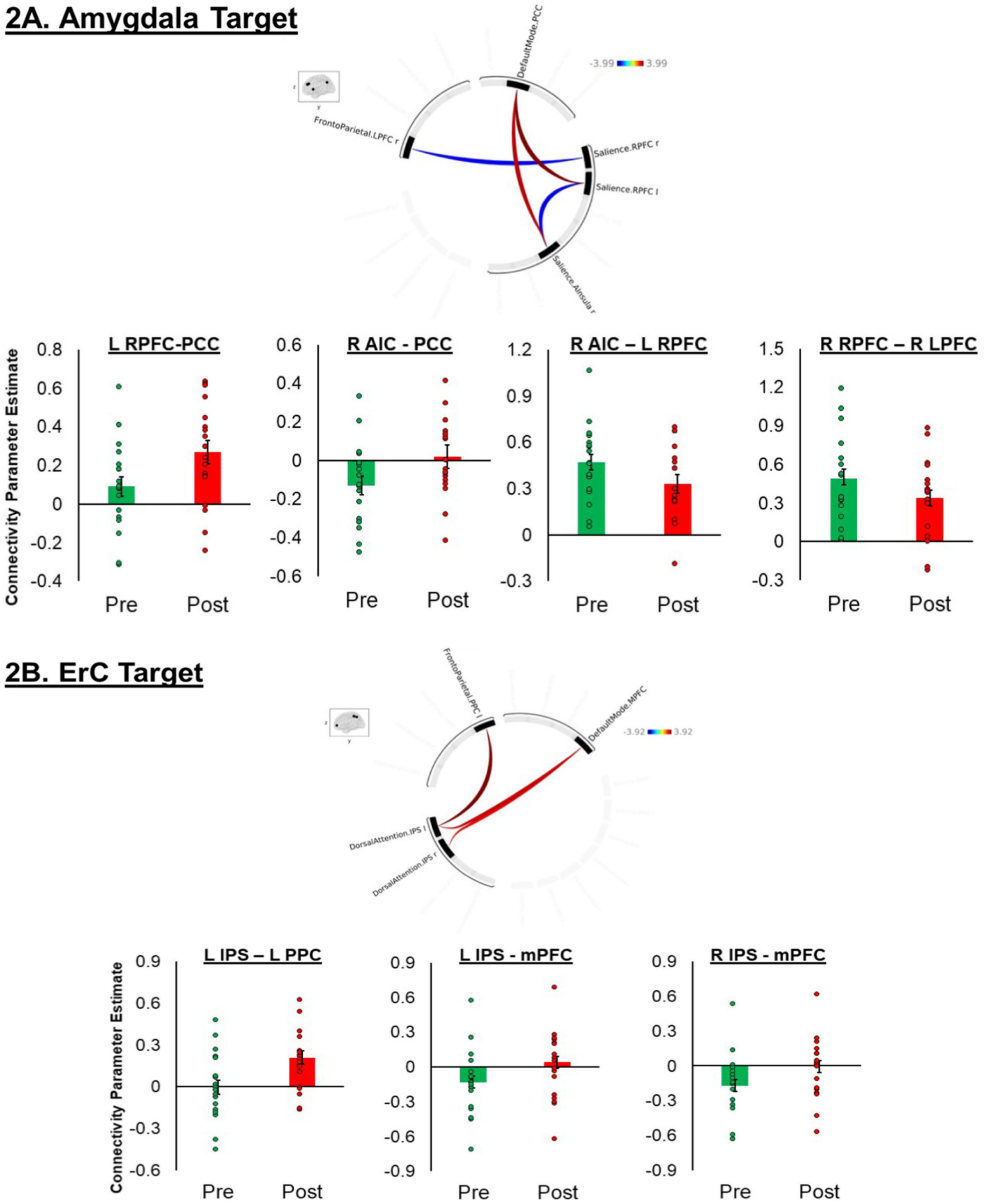
**(A,B)** Circular plots summarizing significant region of interest (ROI)-to-ROI connections in a paired test for the amygdala (Figure 1A) and entorhinal cortex (Figure 1B) targets, highlighting differences at *p* < 0.01. The plot was generated using the CONN toolbox. Significant connections are indicated by colored lines, with red lines representing connections that had greater connectivity post-tFUS compared to pre-tFUS and blue representing connections that had greater connectivity pre-tFUS compared to post-tFUS. The intensity of the colors correlates with the strength of the connections, as shown in the color bar. The inset shows the brain regions involved in the analysis. **(A)** Includes connections between the default mode network (PCC), frontoparietal network (LPFC), and salience network (RPFC and anterior insula). **(B)** Includes connections between the default mode network (mPFC), frontoparietal network (RPFC), and dorsal attention network (IPS). The bar graphs display extracted connectivity parameter estimates from node connections that were found to significantly differ pre-to-post sonication.

The results revealed significant increases in ROI-to-ROI rsFC between the DA network and the FP and DM networks. The following pre-to-post sonication changes in connectivity were observed: (1) increase in the left intraparietal sulcus (IPS; DA network) to the left posterior parietal cortex (PPC; FP network; *t*[17] = 3.92, *p* = 0.0009); (2) increase in the left IPS (DA network) and medial prefrontal cortex (mPFC; DM network; *t*[17] = 3.33, *p* = 0.0037); and (3) increase in the right IPS (DA network) to the mPFC (DM network; *t*[17] = 3.08, *p* = 0.0065) (Figure 2).

### Target comparison

The rsFC parameter estimates for the seven ROI-to-ROI connections (identified above; four amygdala target findings and three ErC target findings) were extracted for both targets, pre- and post-sonication, for the 17 individuals that completed all four scans. These parameter estimates were then entered into a target (amygdala vs. ErC) by time (pre- vs. post-stimulation) repeated measures ANOVA to assess the specificity of each finding.

The results of the seven ANOVAs are presented in Table 2.^1^ There was a significant Target × Time interaction on the right RPFC (SA network) to the right LPFC (FP network) rsFC. Sonication of the right amygdala resulted in a decrease in the right RPFC-right LPFC rsFC (*t*[16] = 2.71, *p* < 0.01); however, sonication of the left ErC produced no change in connectivity between these ROIs (*t*[16] = −1.56, *p* = 0.14). There was also a Target × Time interaction in the left IPS (DA network) to the mPFC (DM network) and the right IPS (DA network) to the mPFC (DM network) rsFC. Sonication of the left ErC resulted in increases in the left IPS to the mPFC (*t*[16] = −3.14, *p* < 0.01) and the right IPS to the mPFC (*t*[16] = −2.99, *p* = 0.01). There were no changes in the left IPS to the mPFC (*t*[16] = 0.72, *p* = 0.48) nor the right IPS to the mPFC (*t*[16] = 0.77, *p* = 0.45) following sonication of the right amygdala.

**Table 2:** Results of the repeated measures analysis of variance for each sonication target.

	Sum of squares	df	Mean square	*F*	*p*-value	Partial eta squared
Amygdala target						
1. Left RPFC–PCC						
Time*	0.665	1, 16	0.665	15.873	0.001	0.498
Target	0.092	1, 16	0.092	1.925	0.186	0.114
Time × Target	0.020	1, 16	0.020	0.622	0.442	0.040
2. Right AIC–PCC						
Time*	0.451	1, 16	0.451	24.085	<0.001	0.601
Target	0.117	1, 16	0.117	3.175	0.094	0.166
Time × Target	0.003	1, 16	0.003	0.119	0.734	0.007
3. Right AIC–Left RPFC						
Time	0.040	1, 16	0.040	2.150	0.162	0.118
Target	0.006	1, 16	0.006	0.318	0.581	0.019
Time × Target	0.067	1, 16	0.067	1.919	0.185	0.107
4. Right RPFC–Right LPFC						
Time	0.006	1, 16	0.006	0.267	0.612	0.016
Target	0.011	1, 16	0.011	0.280	0.604	0.017
Time × Target*	0.217	1, 16	0.217	7.502	0.015	0.319
ErC target						
5. Left IPS–Left PPC						
Time*	0.406	1, 16	0.406	14.303	0.002	0.472
Target	<0.001	1, 16	<0.001	0.001	0.979	<0.001
Time × Target	0.077	1, 16	0.077	2.551	0.130	0.138
6. Left IPS–mPFC						
Time	0.069	1, 16	0.069	1.635	0.219	0.093
Target	0.113	1, 16	0.113	2.062	0.170	0.114
Time × Target*	0.224	1, 16	0.224	7.978	0.012	0.333
7. Right IPS–mPFC						
Time	0.062	1, 16	0.062	2.113	0.165	0.117
Target	0.006	1, 16	0.006	0.076	0.786	0.005
Time × Target*	0.206	1, 16	0.206	6.132	0.025	0.277

*p < 0.05. RPFC, rostral prefrontal cortex; PCC, precuneus cortex; AIC, anterior insular cortex; IPS, intraparietal sulcus; PPC, posterior parietal cortex; mPFC, medial prefrontal cortex.

## Discussion

The current study used resting-state BOLD fMRI data before and after tFUS to examine changes in functional connectivity between nodes of large-scale rs-networks. The results revealed that tFUS of the amygdala (and ErC) changed connectivity within and between rs-networks. Pre-to-post sonication of the right amygdala was found to modulate connectivity within nodes of the SAN and between nodes of the SAN and the DMN and FPN. A decrease in the SAN to FPN connectivity was specific to the amygdala target. Pre-to-post sonication of the left ErC was found to modulate connectivity between the DAN and FPN and DMN. An increase in the DAN to DMN connectivity was specific to the ErC target. These preliminary findings suggest that tFUS may result in neuroplastic changes outside of the focal neural target. Previous studies have shown that low-intensity tFUS does not work via thermal or tissue-damaging mechanisms (Stern et al., 2021; Spivak et al., 2021). The ultrasound waves have a mechanical effect, which changes the membrane potential of neurons in the target region (Tyler, 2011). More broadly, low-intensity tFUS impacts synaptic transmission and changes synaptic efficiency, which may influence the observed changes in functional connectivity beyond the sonication target (Blackmore et al., 2019).

tFUS of the amygdala was associated with changes in connectivity within the SAN and between the SAN and the DMN and FPN. The SAN is involved in the integration of emotionally salient information and makes inferences regarding interoceptive awareness, threat/reward value, and outcome probabilities to appropriately guide approach and avoidance behavior (Seeley et al., 2007; Vytal and Hamann, 2010). Relatedly, the functional integrity within the SAN underlies the subjective experience of affective states (De Witte and Mueller, 2017; Prillwitz et al., 2018). Theory and research further show that the SAN interacts with other large-scale rs-networks and plays a critical role in the dynamic switching between the DMN and the FPN (Sridharan et al., 2008; Goulden et al., 2014). This switching function allows for efficient engagement and disengagement of goal-directed resources. Upon detection of a salient event, the SAN facilitates access to attention and working memory via the FPN (Cai et al., 2021). During rest, the SAN engages the DMN, which supports basic self-referential functions and internally focused attention (Yoon et al., 2019). Regarding the present findings, acute tFUS-related increases in rsFC within the SAN may reflect increased salience processing. Meanwhile, increased SAN to DMN rsFC and decreased SAN to FPN rsFC may signal changes in the SAN switching functions between networks. The decreased rsFC connectivity between the SAN and FPN was unique to the amygdala target, and thus, amygdala inhibition may uniquely decrease SAN input to the FPN. Numerous studies have demonstrated that anxiety and other stress-related disorders are characterized by aberrant SAN function, including deficient network switching (e.g., Li et al., 2023). Additional studies are therefore critically needed to replicate these preliminary findings and elucidate how tFUS-related SAN changes may impact anxiety symptoms.

tFUS of the ErC was used as an active within-subjects comparison. Sonication of the ErC resulted in pre-to-post changes in the DAN to FPN and DMN rsFC. The DAN is comprised of regions in the frontal and parietal cortices (Osher et al., 2019), which become engaged when attention is voluntarily shifted to salient objects and/or locations (Corbetta and Shulman, 2002) and during visual exploration (Corbetta and Shulman, 1998). The DAN also underlies attentional control via top-down influences on the visual cortex (Osher et al., 2019). Anti-correlation between the DAN and DMN is characteristic of typical brain function (Fox et al., 2009; Kelly et al., 2008), which reflects the distinct attentional processes these two networks serve: the DMN mediates internally directed attention, whereas the DAN mediates externally directed attention (Spreng et al., 2017). The magnitude of anti-correlation between the DAN and DMN has been associated with the performance of attention-based tasks in healthy young adults (Hampson et al., 2010), though this association is attenuated by age and seems to differ in patients (Wang et al., 2019; Spreng et al., 2016; Owens et al., 2020). In at least one previous study in adults with depression, positive connectivity between the DAN and DMN was associated with increased memory accuracy for objects (Satz et al., 2022). Interestingly, in a sample of older adults, we found that sonication of the ErC resulted in increases in DAN to DMN rsFC, and this increase was specific to the ErC target. We also found increases in the DAN to FPN rsFC, which are two networks that functionally interact to support perceptual attention (Dixon et al., 2018). Although the functional significance of these acute changes is unknown, it is noteworthy that the DAN, DMN, and FPN are all involved in the cognitive processes that mediate learning and memory—core functions of the ErC target. Thus, the ErC as a target for tFUS in patient groups with amnestic syndromes appears to warrant further exploration.

The pattern of results indicates that some FC changes were more robust following the sonication of one active target but not the other. Sonication of the amygdala target using an inhibition protocol resulted in decreases in rsFC between the SAN and FPN. Meanwhile, sonication of the ErC target using an excitation protocol resulted in increases in the DAN to DMN rsFC. These findings are broadly consistent with our study hypotheses and demonstrate that tFUS can have “downstream” effects that alter rsFC patterns within and between large-scale networks. Other neuromodulation techniques such as deep brain stimulation and transcranial magnetic stimulation have shown similar extended effects (Haslinger et al., 2003; Coenen et al., 2014; Esser et al., 2005). It is noteworthy that changes involving the SAN were exclusively found with tFUS of the amygdala, given that the SAN is the core rs-network involved in salience and emotion processing and is implicated in the pathophysiology of anxiety disorders (Pannekoek et al., 2013). Investigating if and how these SAN-level changes influence affective states and anxiety symptoms is a critical next step, particularly given existing research highlighting the potential clinical utility of tFUS of the amygdala for anxiety (Chou et al., 2024). Meanwhile, the ErC is a brain region involved in memory (Guzman et al., 2013; Eichenbaum, 2000), and tFUS of the ErC modulated DAN rsFC with other attention networks. Attention and memory disturbance, along with associated hypoactivity of the ErC, underlies several neurological disorders, including Alzheimer’s disease (Donix et al., 2013; Maass et al., 2018). Given the dissociative findings observed here, an investigation into the functional significance of DAN-level changes, as they pertain to cognitive processes like memory, may also be a fruitful next step.

The current study had many strengths, including the double-blind, within-subjects crossover design. The study also had several important limitations. First, the study focused on healthy older adults given that there is a need for novel psychiatric and neurological therapeutic strategies for this developmental period. Previous research shows that there are age-related changes in rsFC between large-scale networks, including those investigated in the current study (Grady et al., 2016; Meunier et al., 2009). It is therefore unclear whether the present findings would generalize to younger adults. Second, the sample size was small, although consistent with other published studies involving tFUS in humans (Kuhn et al., 2023; Pasquinelli et al., 2019). It is possible the study was underpowered to detect certain target-specific network-level changes. Relatedly, a total of 171 ROI-to-ROI connections were initially tested, and the findings would not survive stringent correction for multiple comparisons. The length of each resting-state scan was also short, which studies show may decrease the reliability of within-subjects comparisons (Mueller et al., 2015), though our analyses comparing the two baseline target scans showed good reliability. The current findings are therefore considered preliminary and require replication. It is also important to highlight that we targeted contralateral regions (the left ErC and right amygdala) given the close physical proximity of these two regions in the brain. It is possible that the pattern of results may differ if the hemisphere of each target was switched, given prior research on the lateralization of certain networks (Agcaoglu et al., 2015). Finally, the study focused on immediate, acute changes (pre-to-post sonication) and did not measure real-time functional outcomes. Additional studies are needed to determine the duration and durability of the observed findings and if and how network-level changes relate to cognitive and emotional outcomes.

The present findings add to a growing body of literature on the feasibility of tFUS and its acute neural effects. Converging research, including our own, shows that tFUS can target desired areas of the deep brain without engaging nearby structures (Kuhn et al., 2023). We also demonstrate that tFUS can evoke broader changes beyond the focal sonication target. There are many important next steps in this line of work, including the investigation of the functional significance and therapeutic value of these network changes.

## Data Availability

The raw data supporting the conclusions of this article will be made available by the authors, without undue reservation.

## Footnotes

^1^The sample consisted of adults undergoing healthy aging (ages 48–79). To explore the impact of age on the present findings, we split the sample into older (60–79 years; *n =* 8) vs. younger (48–59 years; *n =* 10) adults based on the definition of older adulthood by the WHO (2022). We found that there were no significant two-way (time × age group) or three-way (time × target × age group) interactions involving the age group (all *p*s > 0.28) and thus the pattern of results did not meaningfully differ between the two groups.
